# Chemical Composition and Anticholinesterase Activity of the Essential Oil of Leaves and Flowers from the Ecuadorian Plant *Lepechinia paniculata* (Kunth) Epling

**DOI:** 10.3390/molecules26113198

**Published:** 2021-05-27

**Authors:** María Fernanda Panamito, Nicole Bec, Valeria Valdivieso, Melissa Salinas, James Calva, Jorge Ramírez, Christian Larroque, Chabaco Armijos

**Affiliations:** 1 Departamento de Química, Maestría en Química Aplicada, Universidad Técnica Particular de Loja (UTPL), Calle Marcelino Champagnat s/n, Loja 1101608, Ecuador; vcvaldivieso@utpl.edu.ec (V.V.); masalinas4@utpl.edu.ec (M.S.); jwcalva@utpl.edu.ec (J.C.); jyramirez@utpl.edu.ec (J.R.); 2 Institute for Regenerative Medicine and Biotherapy (IRMB), Université de Montpellier, INSERM, 34295 Montpellier, France; nicole.bec@inserm.fr (N.B.); cjlarroque@gmail.com (C.L.)

**Keywords:** *Lepechinia paniculata*, essential oil, GC-FID/GC-MS, enantiomeric distribution, AChE, BuChE

## Abstract

This work aimed to study the chemical composition, cholinesterase inhibitory activity, and enantiomeric analysis of the essential oil from the aerial parts (leaves and flowers) of the plant *Lepechinia paniculata* (Kunth) Epling from Ecuador. The essential oil (EO) was obtained through steam distillation. The chemical composition of the oil was evaluated by gas chromatography, coupled to mass spectrometry (GC–MS) and a flame ionization detector (GC-FID). The analyses led to the identification of 69 compounds in total, of which 40 were found in the leaves and 29 were found in the flowers of the plant. The major components found in the oil were 1,8-Cineole, β-Pinene, δ-3-Carene, α-Pinene, (*E*)-Caryophyllene, Guaiol, and β-Phellandrene. Flower essential oil showed interesting selective inhibitory activity against both enzymes AChE (28.2 ± 1.8 2 µg/mL) and BuChE (28.8 ± 1.5 µg/mL). By contrast, the EO of the leaves showed moderate mean inhibitory activity against acetylcholinesterase (AChE) and butyrylcholinesterase (BuChE), with IC_50_ values of 38.2 ± 2.9 µg/mL and 47.4 ± 2.3 µg/mL, respectively.

## 1. Introduction

The genus *Lepechinia* belongs to the Lamiaceae family and comprises approximately 43 species distributed from the Southwest USA to Chile [1]. Sesquiterpenes, diterpenes, triterpenes, and flavonoids have been isolated from different species of this genus. Some species are used for their antitumor and insulin-mimetic properties, and to treat uterine infections and stomach pains [2,3].

In the Andean region of Ecuador, the species known as *L. paniculata* is used in traditional medicine to relieve headaches, inflammation, and wound infections, and to cure “mal del aire” and “espanto” [4,5,6].

Regarding the studies of essential oils (EOs) of other *Lepechinia* species from the southern region of Ecuador, in 2002, Malagón et al. [7] identified 54 compounds in *Lepechinia mutica* (Benth) EO collected in “Cerro el Villonaco” (Loja, Ecuador); monoterpene hydrocarbons were the main group of constituents (72%), among which β-Phellandrene (30%), Camphene (13%), Limonene (8%), ∆3-Carene (6%), and α-Pinene (3%) were the most abundant. In another study, Ramírez et al. [2] described the chemical composition, enantiomeric analysis, sensorial evaluation, and antifungal activity of *Lepechinia mutica* EO. Sesquiterpene hydrocarbons (38.50%) and monoterpene hydrocarbons (30.59%) were the most abundant volatiles, while oxygenated sesquiterpenes (16.20%) and oxygenated monoterpenes (2.10%) were minor components [2].

Enantioselective GC–MS analysis is an analytical technique. Its development has been accelerated by the importance of applying its results to the characterization of volatile mixtures such as EOs. In the case of chiral or optically active components, their fragrance and flavor attributes as well as their ability to act as biological mediators are dependent not only on their chemical structures, but fundamentally on their stereochemical properties [8]. It is very common to find enantiomers in EOs due to the metabolic response of plants; thus, there may be compounds for which one enantiomer has toxic activities while the other does not. The enantiomeric composition of the essential oil of *Lepechinia paniculata* leaves and flowers has not been reported in the literature.

These plants have been one of the most important sources for the search of compounds with inhibitory activity against acetylcholinesterase (AChE) and butyrylcholinesterase (BuChE) [9]. Acetylcholine (ACh) is an essential neuromodulator involved in neuronal influx transmission [10] and, more specifically, in memory connection [11]. Acetylcholine degradation is mediated by specific enzymes such as AChE and, to a lesser extent, BuChE. The low concentration of this neuromodulator is a key factor in Alzheimer’s disease. Therefore, many drugs that inhibit acetylcholine degradation are on the market, but they have limited efficacy [12,13]. Our objective was to look in plants selected based on the knowledge of traditional medicine for new compounds that could regulate acetylcholine degradation. Studying the inhibition potential of plant extracts and EOs on acetyl- and butyrylcholinesterase activity is thus an essential step in the discovery of new strategies to improve the quality of life of Alzheimer’s patients. According to the World Health Organization (WHO), Alzheimer’s disease (AD) is currently the leading cause of dementia in the world, responsible for 60–70% of cases [14,15].

This paper reports the chemical composition, cholinesterase inhibitory activity, and the enantiomeric analysis of the EO from the aerial parts (leaves and flowers) of *Lepechinia paniculata*. This study is a part of our ongoing research on the valorization of aromatic plants from Ecuador.

## 2. Results

### 2.1. Extraction Performance

Three extractions were made of on both the leaves and the flowers of *Lepechinia paniculata* by steam distillation. The essential oil extraction yield was 0.49 ± 0.25% for the leaves and 0.15 ± 0.01% for the flowers. 

### 2.2. Chemical Composition

Analyses were performed using gas chromatography coupled to mass spectrometry (GC–MS) and a flame ionization detector (GC–FID) with a non-polar DB-5MS column and a polar HP-INNOWax column. The identification of the compounds was carried out with ChemStation software coupled to the gas chromatograph, which was also used to carry out the experimental comparison of the calculated linear retention indices (LRI^Exp^) with those of the mass spectra from the literature (LRI^Ref^).

Table 1 shows the result of the EO chemical composition of *Lepechinia paniculata*. In the leaves, 40 compounds were identified that represented 98.34% of the total composition on the DB-5MS chromatographic column and 98.40% of the total composition on the HP-INNOWax column. Similarly, in the EO of the flowers, 29 compounds were identified that represented 97.62% of the total composition on the DB-5MS column and 98.43% of the total composition on the HP-INNOWax column.

**Figure 1:**
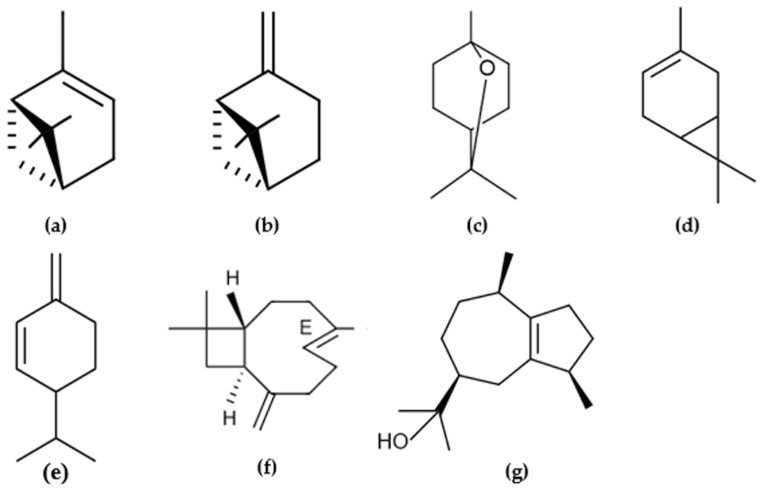
Structures of selected constituents (contents > 4%) identified in essential oils (leaves and flowers) of *Lepechinia paniculata*: (**a**) α-Pinene; (**b**) β-Pinene; (**c**) 1,8-Cineole; (**d**) δ-3-Carene; (**e**) β-Phellandrene; (**f**) (*E*)-Caryophyllene; (**g**) Guaiol.

The most representative compounds found on the DB-5MS column were α-Pinene (18.37% in the leaves and 6.52% in the flowers), δ-3-Carene (4.14% in the leaves and 10.63% in the flowers), (*E*)-Caryophyllene (15.39% in the leaves and 9.88% in the flowers), β-Phellandrene (8.62% in the leaves and 4.50% in the flowers), Guaiol (8.58% in the leaves and 4.46% in the flowers), 1,8-Cineole (7.66% in the leaves and 5.70% in the flowers), and β-Pinene (5.67% in the leaves and 10.90% in the flowers) (Table 1, Figure 1).

**Figure 2:**
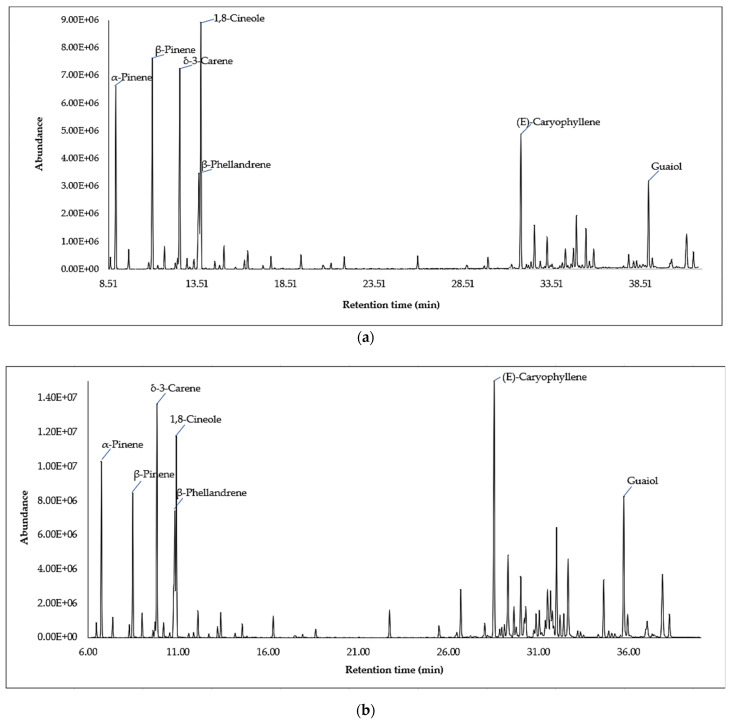
Typical gas chromatogram (GC-MS on DB-5MS) of essential oil from *Lepechinia paniculata*: (**a**) leaves; (**b**) flowers.

A typical chromatogram of *Lepechinia paniculata* essential oil is shown in Figure 2.

### 2.3. Enantioselective Analysis

Enantiomer components and their enantiomer excesses (*ee*) in *L. paniculata* EO obtained from the leaves and flowers were determined by enantioselective GC–MS analysis. Three pairs of enantiomers were detected for the EO of leaves, and two pairs were detected for the EO of flowers, as shown in Table 2. The order of enantiomeric elution was established by the separated injections of the enantiomerically pure standards.

### 2.4. Cholinesterase Inhibition Assay

The *Lepechinia paniculata* EOs of flowers showed quite remarkable inhibitory activity against both the enzymes AChE (IC_50_ = 28.2 ± 1.8 µg/mL) and BuChE (IC_50_ = 28.8 ± 1.5 µg/mL). By contrast, the EO from the leaves showed a moderate mean inhibitory concentration against AChE (IC_50_ = 38.2 ± 2.9 µg/mL) and against BuChE (IC_50_ = 47.4 ± 2.3 µg/mL).

## 3. Discussion

Regarding the chemical composition of *Lepechinia paniculata* EO, a previous study reported the sesquiterpenes Aromadendrene (24.64%) and Viridiflorene (12.37%) as well as the monoterpene β-Phellandrene (7.72%) as major compounds [19]. However, the current study confirmed that the EO from *Lepechinia paniculata* was characterized by the following major compounds: α-Pinene, (*E*)-Caryophyllene, β-Phellandrene, Guaiol, 1,8-Cineole, and β-Pinene. These results may help correctly distinguish the species *L. paniculata* from other *Lepechinia* spp. since the taxonomic identification of *Lepechinia* species is complicated by their similarity.

Regarding the enantioselective GC–MS analysis, (+)-α-Pinene had a high *ee* compared with the enantiomeric excesses of (+)-δ-3-Carene in the essential oil of flowers. By contrast, for the EO of leaves, the enantiomeric excesses of (+)-δ-3-Carene were moderate, and those of (−)-α-Pinene and (−)-Terpinolene were low. These results further confirm that chiral secondary metabolites are often present in plants as enantiomeric mixtures. The determination of the enantiomeric purity of a natural or synthetic compound is of great importance for different areas because each enantiomer of a molecule has different properties [8,18].

Finally, the development of new acetylcholinesterase (AChE) and butyrylcholinesterase (BuChE) inhibitors represents a viable approach to alleviate Alzheimer’s disease [31]. The inhibition of BuChE and AChE is of great interest for the study of the treatment and slowing down of Alzheimer’s disease [12,32] and other neurodegenerative diseases. The inhibitory activity of *Lepechinia paniculata* EO for the two enzymes evaluated has not been previously described in the literature, so new studies are necessary to establish its potential pharmacological use.

## 4. Materials and Methods

### 4.1. Plant Material

Aerial parts of *L. paniculata* were collected in the flowering stage in March and April 2019 in the El Tablon sector in the Loja province of southern Ecuador, at an altitude of 1000 m.a.s.l. The geographical coordinates were 3°30′41.9″ S 79°09′18.2″ W, 704948.6E -9611806.7N -3.511648, -79.155052. Nixon Cumbicus identified the plant in the Herbarium of the Universidad Técnica Particular de Loja (HUTPL). The plant collection was authorized under governmental permission (MAE-DBN-2016-065).

### 4.2. Isolation of Essential Oil

The leaves and flowers were separated, and steam distilled immediately after collection for 3 h using a Clevenger-type apparatus in the Universidad Técnica Particular de Loja (UTPL). The essential oil was then separated from the aqueous phase and dried over anhydrous sodium sulphate, filtered, and stored in brown vials at 4 °C until the analysis. This procedure was repeated three times for each EO.

### 4.3. Chemical Composition of Essential Oil

For the qualitative determination of the components, gas chromatography coupled to mass spectrometry (GC–MS) was used, and for the quantitative analysis, gas chromatography coupled to a flame ionization detector was used (GC–FID).

The analyses for GC–MS were carried out on an Agilent Technologies gas chromatograph 6890N series gas chromatograph coupled to an Agilent mass detector, series 5973 Inert (Santa Clara, CA, USA), electronic impact (70 eV), with a series 7683 autoinjector. The gas chromatograph was coupled with MSD-ChemStation software to recognize the compounds of the volatile fraction of the *L. paniculata* species.

Two types of chromatographic columns were used: a non-polar capillary column, DB-5MS (Agilent Technologies) (5%-phenyl-methylpolysiloxane stationary phase, 30 m × 0.25 mm i.d. × 0.25 μm film thickness; J; W Scientific, Folsom, CA, USA), and a polar capillary column, HP-INNOWax (Agilent Technologies) (polyethylene glycol, 30 m × 0.25 mm i.d. × 0.25 μm film thickness; J; W Scientific, Folsom, CA, USA), both using helium as carrier gas (1.00 mL/min in constant flow mode). The injection system operated in split mode (40:1) at 220 °C. The GC oven temperature was kept at 60 °C, then increased to 250 °C with a gradient rate of 3 °C/min. The ion source temperature was 250 °C. A quantity of 1 μL of a solution of the oil in CH_2_Cl_2_ (1:100 *v*/*v*) was injected.

The analyses for GC–FID were performed using an Agilent Technologies chromatograph 6890N series (Santa Clara, CA, USA) coupled to an FID 7683 series (Little Falls, DE, USA) using the DB-5MS and HP-INNOWax columns. The quantification (expressed as a relative percentage) of each identified compound was performed by comparing the area of the corresponding GC peak to the total area of identified peaks (Table 1) without applying any correction factors. The average values and standard deviations were calculated from the results of three injections. The EO samples were prepared and analyzed under the same conditions as the GC–MS analysis.

### 4.4. Enantiomeric Analysis

The enantiomeric distribution and enantiomeric excess of some chiral metabolites were determined on a cyclodextrin-based chiral stationary phase MEGA-DEX-DET from Mega (Legnano, MI, Italy), comparing the retention time of separated enantiomers with enantiomerically pure standards.

### 4.5. Cholinesterase (ChE) Inhibition Assay

The inhibition of two cholinesterase enzymes (ChEs), acetylcholinesterase (AChE, from *Electrophorus electricus*, Sigma-Aldrich, SRE020, St Louis, MO, USA) and butyrylcholinesterase (BuChE, from equine serum, Sigma Aldrich, SRE020, St. Louis, MO, USA), both of which are acetylcholine-hydrolyzing enzymes [32], was determined by a colorimetric procedure reported by Ellman et al. (1961) [33]. The volume used for the inhibition analysis contained 200 μL of phosphate-buffered saline (pH 7.4), 1.5 mM of DTNB, and the EO sample dissolved in DMSO (1% *v*/*v*). The two enzymes AChE and BuChE were dissolved in phosphate-buffered saline (pH 7.4), and 24 mU/mL was taken for each test performed. After 10 min of preincubation, the acetylcholine iodide substrate (1.5 mM) was added to start the reaction. After 30 min at 30 °C, the 96-well microplates were read on a PherastarFS detection kit (BMG Labtech). The measurements were made in triplicate for the EO of leaves and flowers. IC_50_ values were calculated using the GNUPLOT online program (www.ic50.tk, www.gnuplot.info, accessed on 21 January 2021). The reference inhibitor used was donepezil, with IC_50_ = 100 nM for AChE and 8500 nM for BuChE. For the analysis, the false-positive results (>100 µg/mL), which may have occurred due to the presence of amine compounds or aldehydes, were excluded [34].

## 5. Conclusions

The analysis on the DB-5MS capillary column showed that the EOs of *L. paniculata* leaves mainly consisted of monoterpene hydrocarbons (48.80%), followed by sesquiterpene hydrocarbons (25.59%), and the EOs of the flowers mainly consisted of sesquiterpene hydrocarbons (40.07%), followed by monoterpene hydrocarbons (39.00%).

The major chemical compounds identified in the essential oils from the leaves and flowers were 1,8-Cineole, β-Phellandrene, β-Pinene, δ-3-Carene, α-Pinene, (*E*)-Caryophyllene, and Guaiol. The identified compounds belong for the most part to the chemical group of hydrocarbon monoterpenes.

As a complementary contribution to the chemical composition study, the enantiomeric distribution of the EO was analyzed, identifying the following pairs of enantiomers: (A) (+)-α-Pinene, (−)-α-Pinene; (B) (+)-δ-3-Carene, (−)-δ-3-Carene; (C) (+)-Terpinolene, (−)-Terpinolene.

The EO of the leaves of *Lepechinia paniculata* showed moderate inhibitory activity against both cholinesterase enzymes evaluated, with IC_50_ values of 38.2 ± 2.9 µg/mL against AChE and 47.4 ± 2.3 µg/mL against BuChE, whereas in the EO of the flowers, the inhibitory activity was much more marked, with IC_50_ values of 28.2 ± 1.8 µg/mL against AChE and 28.8 ± 1.5 µg/mL against BuChE.

Finally, the results obtained in the study of the essential oil from the leaves and flowers of *Lepechinia paniculata* constitute the first report on the AChE and BuChE activity for this species.

## Figures and Tables

**Table 1:** Chemical composition of the leaves and flowers of *Lepechinia paniculata* essential oil.

		DB-5MS Column				HP-INNOWax Column				
				Leaves	Flowers			Leaves	Flowers	
N°	Compound	LRI^Exp^	^1^ LRI^Ref^	% ± σ	% ± σ	LRI^Exp^	LRI^Ref^	% ± σ	% ± σ	Ref. LRI
1	α-Thujene	920	924	0.57 ± 0.28	0.53 ± 0.04	-	-	-	-	-
2	α-Pinene	927	932	18.37 ± 0.45	6.52 ± 0.73	1058	1066	11.10 ± 0.08	8.70 ± 0.31	[16]
3	Camphene	942	946	0.59 ± 0.02	0.86 ± 0.39	1082	1084	1.04 ± 0.01	1.21 ± 0.04	[16]
4	Sabinene	966	969	0.69 ± 0.14	0.59 ± 0.06	1122	1132	0.44 ± 0.00	0.69 ± 0.03	[17]
5	β-Pinene	970	974	5.67 ± 0.36	10.90 ± 0.58	1112	1118	14.11 ± 0.07	16.27 ± 0.58	[17]
6	1-Octen-3-ol	976	974	0.11 ± 0.12	-	1455	1451	0.09 ± 0.00	-	[18]
7	Myrcene	984	988	0.60 ± 0.34	1.09 ± 0.19	1168	1166	0.29 ± 0.25	-	[18]
8	α-Phellandrene	1004	1002	0.42 ± 0.53	0.42 ± 0.05	1165	1160	2.05 ± 0.01	1.60 ± 0.04	[18]
9	δ-3-Carene	1006	1008	4.14 ± 0.79	10.63 ± 0.87	1149	1159	12.44 ± 0.06	10.97 ± 0.35	[17]
10	α-Terpinene	1014	1014	2.18 ± 0.42	0.46 ± 0.10	1179	1188	0.48 ± 0.01	0.50 ± 0.01	[17]
11	Limonene	-	-	-	-	1201	1203	2.40 ± 0.01	2.14 ± 0.07	[17]
12	ρ-Cymene	1022	1020	3.00 ± 0.55	-	1270	1280	0.42 ± 0.00	-	[17]
13	β-Phellandrene	1028	1025	8.62 ± 0.17	4.50 ± 0.97	-	-	-	-	-
14	1,8-Cineole	1030	1026	7.66 ± 0.37	5.70 ± 0.35	1210	1213	18.73 ± 0.12	7.16 ± 0.36	[17]
15	(*Z*)-β-Ocimene	-	-	-	-	1241	1213	0.15 ± 0.00	-	[17]
16	(*E*)-β-Ocimene	1046	1044	0.33 ± 0.02	-	1254	1253	0.41 ± 0.00	0.83 ± 0.00	[16]
17	γ-Terpinene	1056	1054	3.37 ± 0.22	1.24 ± 0.45	1245	1255	1.04 ± 0.01	0.89 ± 0.03	[17]
18	ρ-Mentha-2,4(8)-diene	1079	1085	-	0.40 ± 0.02	-	-	-	-	-
19	Terpinolene	1082	1086	0.25 ± 0.04	0.86 ± 0.07	1282	1290	0.84 ± 0.01	0.70 ± 0.02	[17]
20	1-Octen-3-yl acetate	1108	1110	-	0.71 ± 0.19	1384	1381	0.70 ± 0.01	0.67 ± 0.03	[18]
21	Camphor	1143	1141	0.26 ± 0.09	-	1506	1515	0.73 ± 0.03	-	[19]
22	δ-Terpineol	1169	1162	0.15 ± 0.08	-	-	-	-	-	-
23	Terpinen-4-ol	1178	1174	0.09 ± 0.04	-	1600	1590	0.29 ± 0.01	-	[18]
24	α-Terpineol	1193	1186	0.30 ± 0.08	-	1697	1700	-	0.44 ± 0.02	[20]
25	*n*-Decanal	1206	1201	0.23 ± 0.20	-	-	-	-	-	-
26	Isobornyl acetate	1282	1283	2.94 ± 0.28	-	1576	1575	0.68 ± 0.02	-	[16]
27	Bornyl acetate	1282	1284	-	0.90 ± 0.31	1576	1570	-	1.30 ± 0.03	[17]
28	α-Cubebene	1344	1348	-	0.49 ± 0.03	1452	1460	0.11 ± 0.01	0.41 ± 0.01	[21]
29	α-Copaene	1371	1374	1.92 ± 0.33	2.06 ± 0.28	1482	1483	0.43 ± 0.01	2.02 ± 0.16	[16]
30	α-Gurjunene	1402	1409	0.37 ± 0.05	0.47 ± 0.13	1519	1520	0.12 ± 0.01	0.53 ± 0.02	[16]
31	Linalool	-	-	-	-	1554	1553	0.12 ± 0.10	-	[17]
32	(*E*)-Caryophyllene	1414	1417	15.39 ± 0.58	9.88 ± 0.58	1588	1586	7.27 ± 0.11	8.01 ± 0.10	[16]
33	β-Gurjunene	1425	1431	-	0.40 ± 0.01	-	-	-	-	-
34	Aromadendrene	1433	1439	1.78 ± 0.37	4.40 ± 0.64	1596	1589	2.48 ± 0.04	3.99 ± 0.07	[19]
35	α-Guaiene	-	-	-	-	1604	1583	-	0.39 ± 0.01	[22]
36	*trans*-Muurola-3,5-diene	1441	1451	-	0.88 ± 0.28	-	-	-	-	-
37	*allo*-Aromadendrene	-	-	-	-	1632	1633	0.13 ± 0.00	-	[16]
38	α-Humulene	1450	1452	1.21 ± 0.16	2.10 ± 0.48	1658	1657	1.74 ± 0.02	1.79 ± 0.04	[16]
39	*cis*-Cadina-1(6),4-diene	1455	1461	-	0.72 ± 0.39	-	-	-	-	-
40	*cis*-Muurola-4(14),5-diene	1457	1465	-	0.92 ± 0.15	-	-	-	-	-
41	γ-Muurolene	1470	1478	-	0.83 ± 0.05	1680	1667	-	0.95 ± 0.02	[16]
42	α-Amorphene	-	-	-	-	1679	1679	0.35 ± 0.00	-	[23]
43	γ-Curcumene	1475	1481	1.25 ± 0.23	1.64 ± 1.02	1685	1688	1.83 ± 0.02	-	[16]
44	*cis*-β-Guaiene	1486	1492	-	3.59 ± 0.61	1686	1667	-	4.09 ± 0.09	[24]
45	Isoborneol	-	-	-	-	1697	1698	0.87 ± 0.00	-	[25]
46	α-Selinene	-	-	-	-	1712	1722	-	0.29 ± 0.01	[26]
47	*ar*-Curcumene	1478	1479	-	0.69 ± 0.12	1771	1771	0.19 ± 0.01	-	[16]
48	β-Selinene	1483	1489	0.04 ± 0.04	-	1706	1708	-	0.36 ± 0.00	[22]
49	Viridiflorene	1486	1496	0.12 ± 0.02	4.97 ± 0.03	1725	1710	0.12 ± 0.00	2.92 ± 0.06	[16]
50	α-Zingiberene	1490	1493	1.28 ± 0.45	-	1730	1737	2.40 ± 0.17	-	[16]
51	Epizonarene	1492	1501	-	2.03 ± 0.73	1704	1688	-	1.07 ± 0.02	[16]
52	(*E*.*E*)-α-Farnesene	1503	1505	1.77 ± 0.08	-	1751	1754	2.99 ± 0.01	-	[27]
53	δ-Amorphene	1508	1511	0.21 ± 0.15	-	-	-	-	-	-
54	γ-Cadinene	1514	1513	0.26 ± 0.23	0.72 ± 0.13	-	-	-	-	-
55	δ-Cadinene	1514	1522	-	3.28 ± 0.59	1751	1750	-	4.35 ± 0.12	[28]
56	α-Curcumene	-	-	-	-	1771	1770	-	0.72 ± 0.01	[19]
57	*cis*-Calamenene	-	-	-	-	1822	1816	-	0.31 ± 0.00	[25]
58	Palustrol	1564	1567	0.22 ± 0.22	1.03 ± 0.72	1915	1915	0.53 ± 0.01	0.51 ± 0.13	[16]
59	Spathulenol	1571	1577	0.12 ± 0.12	0.46 ± 0.17	2117	2118	0.34 ± 0.01	0.73 ± 0.01	[16]
60	Caryophyllene oxide	1576	1582	0.14 ± 0.01	-	1966	1967	0.35 ± 0.00	0.63 ± 0.00	[16]
61	Guaiol	1593	1600	8.58 ± 0.16	4.46 ± 0.53	2087	2094	3.95 ± 0.06	4.52 ± 0.11	[27]
62	Ledol	1598	1602	-	0.92 ± 0.29	2016	2017	0.44 ± 0.03	1.34 ± 0.02	[16]
63	Viridiflorol	-	-	-	-	2065	2065	-	0.26 ± 0.01	[22]
64	Globulol	-	-	-	-	2066	2051	0.13 ± 0.00	-	[19]
65	10-epi-γ-Eudesmol	1627	1622	-	0.93 ± 0.20	-	-	-	-	-
66	α-Eudesmol	1650	1652	2.72 ± 0.56	2.73 ± 0.64	2214	2229	1.26 ± 0.02	1.56 ± 0.04	[29]
67	Bulnesol	1660	1670	0.41 ± 0.32	0.71 ± 0.10	2206	2205	0.71 ± 0.01	0.78 ± 0.03	[23]
68	γ-Eudesmol	-	-	-	-	2170	2178	0.26 ± 0.03	0.52 ± 0.07	[27]
69	β-Eudesmol	-	-	-	-	2223	2231	0.86 ± 0.01	1.32 ± 0.03	[27]
Monoterpene hydrocarbons		(%)		48.80	39.00			47.22	44.47	
Oxygenated monoterpenes		(%)		9.04	6.41			21.44	8.32	
Sesquiterpene hydrocarbons		(%)		25.59	40.07			20.15	32.19	
Oxygenated sesquiterpenes		(%)		12.20	11.24			8.82	12.17	
Others		(%)		3.05	0.90			0.77	1.30	
TOTAL IDENTIFIED		(%)		98.34	97.62			98.40	98.43	

^1^ LRI^Ref^, linear retention index obtained from the literature [30]; LRI^Exp^, linear retention index calculated against n-alkanes C9–C24; % ± σ, percentage and standard deviation of each compound determined from the GC–FID chromatogram.

**Table 2:** Enantiomeric composition of *L. paniculata* essential oil.

	LEAVES				FLOWERS	
Compound	RT ^1^ (min)	LRI ^2^	Enantiomeric Distribution	Enantiomeric Excess	Enantiomeric Distribution	Enantiomeric Excess
			%	%	%	%
(+)-α-Pinene	5.43	928	35.15	29.70	99.02	98.05
(−)-α-Pinene	5.55	930	64.85		0.98	
(+)-δ-3-Carene	8.48	985	94.99	89.98	99.86	99.72
(−)-δ-3-Carene	8.79	991	5.01		0.14	
(+)-Terpinolene	12.93	1068	64.58	29.15	-	-
(−)-Terpinolene	13.14	1072	35.42			

^1^ RT: retention time. ^2^ LRI: linear retention index calculated on MEGA-DEX-DET chiral stationary phase.

## Data Availability

The data presented in this study are available in this article.

## Sample Availability

Samples of the compounds are available from the authors.
